# Global warming in the context of 2000 years of Australian alpine temperature and snow cover

**DOI:** 10.1038/s41598-018-22766-z

**Published:** 2018-03-13

**Authors:** Hamish McGowan, John Nikolaus Callow, Joshua Soderholm, Gavan McGrath, Micheline Campbell, Jian-xin Zhao

**Affiliations:** 1 Atmospheric Observations Research Group, School of Earth and Environmental Sciences, The University of Queensland, Brisbane, 4072 Australia; 2 UWA School of Agriculture and Environment, The University of Western Australia, Perth, 6009 Australia; 3 Radiogenic Isotope Facility, School of Earth and Environmental Sciences, The University of Queensland, St, Brisbane, 4072 Australia

## Abstract

Annual resolution reconstructions of alpine temperatures are rare, particularly for the Southern Hemisphere, while no snow cover reconstructions exist. These records are essential to place in context the impact of anthropogenic global warming against historical major natural climate events such as the Roman Warm Period (RWP), Medieval Climate Anomaly (MCA) and Little Ice Age (LIA). Here we show for a marginal alpine region of Australia using a carbon isotope speleothem reconstruction, warming over the past five decades has experienced equivalent magnitude of temperature change and snow cover decline to the RWP and MCA. The current rate of warming is unmatched for the past 2000 years and seasonal snow cover is at a minimum. On scales of several decades, mean maximum temperatures have undergone considerable change ≈ ± 0.8 °C highlighting local scale susceptibility to rapid temperature change, evidence of which is often masked in regional to hemisphere scale temperature reconstructions.

## Introduction

Historical documents, written and oral histories give insight to impacts that shifts in global climate had on empires, economies and the environment over the past two thousand years. This includes periods of relative warmth at the height of the Roman Empire known as the Roman Warm Period (RWP)^1,2^ and the Viking expansion during the Medieval Climate Anomaly (MCA), contrasting with social and economic disruption during the cold of the Little Ice Age (LIA)^3–5^. The gradual transitions to these climate states are in contrast to abrupt changes in climate linked to major volcanic eruptions such as Tambora, Samalas and Krakatoa^6,7^ lasting several years, the solar forced Maunder Minimum^8,9^, and the cyclic climate patterns of El Niño-Southern Oscillation, North Atlantic Oscillation and Indian Ocean Dipole^10–12^. Placing recent anthropogenic global warming in the context of the rate and magnitude of such natural climate variability is essential to understand likely impacts of future climate change. Nowhere is this more important than in regions where subtle changes in climate may result in disproportionate and rapid environmental change. Marginal alpine areas are at particular risk in this context as small changes in surface – atmospheric energetics may cause dramatic decreases in snow cover affecting alpine hydrology, snow dependent ecosystems, hydroelectric power generation and tourism^13^.

The ability to reconstruct climate at nearly annual resolution has been limited by paleo-records that are either remote-from-source (i.e. glacial records from distant areas), or terrestrial records that have relatively high signal to noise and poor temporal resolution^14^. For marginal alpine areas that are sensitive to climate variability and change there is a distinct lack of high resolution paleo-records in the Southern Hemisphere^11,12,14–16^. As a result, the context of anthropogenic global warming and impacts in these regions relative to natural climate variability is poorly resolved.

The marginal alpine snowpack of the Australian Alps is critical to water resources of south-eastern Australia due to the concentration of population; agricultural production, hydroelectric power generation and seasonal snow sport industry, worth an estimated AUD $1.5 billion annually^17^. Maximum snow depth in the Australian Alps has declined by up to 15% since the 1960s, particularly in spring, and model predictions suggest this trend will continue along with a reduction in length of snow season in response to global warming^18–20^. Seasonal snow is also influenced by teleconnection forcing with snow depths shown to strongly decline during El Niño or positive Southern Annular Mode (SAM) events^17^. Placing these findings in a longer deep-time context has been hindered by short observational hydroclimate records which like other regions with high dependency on snow and snowmelt runoff such as California, seldom exceed 80 years^21^. Here we present a 2000 year reconstruction of temperature and snow cover for the Australian Alps based on a near annual resolution δ^13^C speleothem record. The record establishes a context against which changes in hydroclimate thought to be in response to global warming can be gauged, against climatic events in the recent geologic past including the Little Ice Age and Medieval Climate Anomaly.

## Developing an alpine record of temperature and snow cover

**Figure 1:**
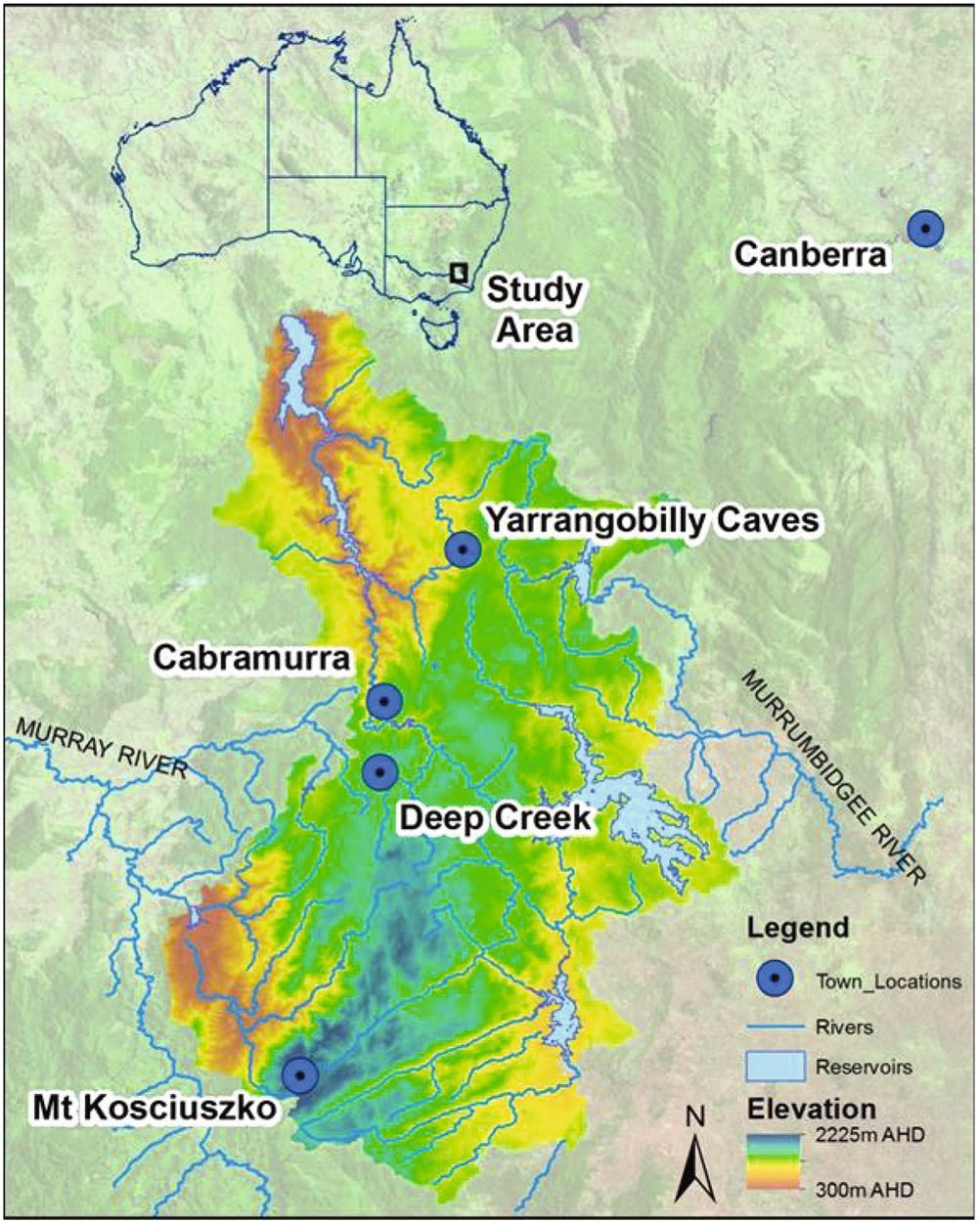
Location map of study area with places referred to in text indicated. Figure created using ESRI ArcGIS 10.3.1 (Version: 10.3.1.4959, http://www.esri.com/arcgis/about-arcgis), with geospatial data provided by Snowy Hydro Ltd. (river, reservoir and elevation) and other data from Geoscience Australia under a Creative Commons 4.0 International Licence.

The Yarrangobilly Caves karst complex (950 to 1,050 m above Australian Height Datum) located in the northern section of Kosciusko National Park offers a unique potential for preservation of long and detailed records of alpine hydroclimate (Fig. 1). Annual precipitation ranges from 760–2800 mm with the majority of precipitation falling in winter, primarily as snow above 1400 m. A trend has emerged since the 1950s toward increasing warm season (November – March) rainfall with moisture sourced from the tropical seas north of Australia^22^.

The local climate means this site is at the contemporary boundary of water/energy limited systems (~1000 m ASL), i.e. where subtle changes in climate can cause a switch from energy (demand) to supply (precipitation) limited water balances^23^. As a result, soil biotic activity and the decomposition of soil organic matter in this area are highly sensitive to changes in temperature, and to a lesser amount precipitation. In cool-climate settings such as the Australian Alps, soil respiration δ^13^C has been shown to display a negative linear relationship to temperature, as temperature affects utilisation of carbon with different δ^13^C signatures^24–26^. The δ^13^C record preserved in underlying karst formations can therefore contain excellent preservation of climate variability in conjunction with the frequently used δ^18^O record, taken as proxy for meteoric water temperature, and therefore ambient air temperature^25^.

We collected stalagmite (JC001) under licence from the “Grotto of Oddities”, Yarrangobilly Caves with topographic survey and Electrical Resistivity Tomography (ERT) hydrogeophysics confirming this cave is overlain by only 6–8 m of epikarst within which there is a locally wet reservoir overtop^27^. Drip water monitoring over 36 months and modelling of epikarst hydrogeology found JC001 was fed by a perennial, seasonally responsive seepage flow^27,28^. Drip modelling over the period of the local instrument record (1978–2014) confirmed seasonally responsive perennial drip behaviour including during the Millennium drought (2001–2009), one of the driest periods of the last 500 years^29^. Monitoring of cave temperature found that it followed an annual cycle of between 10 to 11 °C.

One thousand and forty six sub-samples were milled and analysed from the top 40 mm of the stalagmite and 697 samples from the following 40 mm representing every second sample. These samples underwent stable isotope analysis (δ^18^O and δ^13^C) with the correlation between δ^13^C and δ^18^O for the whole data set found to be very low (r^2^ = −0.12), indicating that JC001 formed in isotopic equilibrium^30,31^.

Eleven samples underwent uranium-series dating from which the age model for JC001 was developed (Supplementary Table S1). The ensemble age model (Supplementary Figure S1) gives an average temporal resolution per sample of 1.03 yrs between 932 CE to 2012 CE and 1.6 yrs between 931 CE to 188 BCE. As such the record is unprecedented as it was collected from a responsive and close to surface location; is fed by a seasonally-responsive perennial drip; is likely to preserve a seasonal to annual paleo-record sampled at near annual resolution; was collected from a site at the energy-water balance limitation transition^23^; and from a region where the hydroclimate is strongly influenced by global-scale teleconnections^32,33^.

Annual mean maximum temperature (Tmax) data was obtained from the Australian Water Availability Project (AWAP)^34^ for 1911 to 2015. AWAP data is topographically resolved with a spatial resolution of 0.05° × 0.05°. We extracted data for the grid point (−35.7°S, 148.5°E) located nearest to Yarrangobilly Caves. Snow depth and duration data was obtained from Snowy Hydro Ltd. for Deep Creek located near Cabramurra (36.035°S, 148.374°E) at 1620 m (1957–2013) (Fig. 1) for the number of days each winter snow cover exceeded 50 cm.

Linear models were developed based on the best fit of five year simple moving averages of Tmax^35^ and number of days each winter snow cover exceeded 50 cm to δ^18^O and δ^13^C data from JC001, to reconstruct air temperature and snow cover for the period 188 BCE – 2012 CE. While temperature-elevation effects are known to strongly influence the precipitation δ^18^O isotope record in the region^36^, the speleothem δ^18^O relationship to environmental variables was found to switch on approximate centennial scale, with the most recent change in the early 1950s^22^. This corresponds directly to the de-coupling of the δ^18^O record from the more conservative δ^13^C paleo-record and temperature data from the region. Change in the relative contributions of precipitation from Southern Ocean weather systems to those from tropical seas northwest of Australia^22^ is believed to have caused the decoupling of the δ^18^O record from local temperature records. Accordingly, the use of δ^18^O to reconstruct paleoclimate and paleo-hydrology for this region is not possible. As a result, reconstructions of Tmax and snow cover were developed from the linear relationships between the more conservative JC001 δ^13^C record and AWAP Tmax and measured snow cover (Supplementary Figure S2).

**Figure 2:**
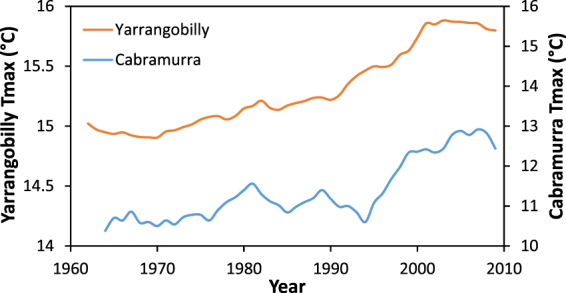
Reconstructed Yarrangobilly Caves Tmax calculated from the δ^13^C JC001 stalagmite record and AWAP Tmax transfer function (Supplementary Figure S2), and observed Cabramurra Tmax (5 yr. simple running averages; 1962 to 2009 correlation between both datasets, R^2^ = 0.81).

A comparison of reconstructed Yarrangobilly Caves Tmax calculated from the δ^13^C JC001 stalagmite record and AWAP Tmax compared against observed annual measured Tmax for the village of Cabramurra is presented in Fig. 2. The fidelity of our reconstructed temperature record is highlighted through comparison with the observational record from Cabramurra. Both records show a warming trend and decreased rate of warming early this century reflecting the impact of the 2010–11 La Niña in moderating the warming trend of the previous fifty years (Fig. 2)^37–39^. The temperature offset of 3 °C between the two records is the result of the difference in elevation between sites with the cooler Cabramurra record taken at around 350 m higher in elevation than Yarrangobilly Caves (Fig. 1).

### 2000 years of alpine temperature and snow cover variability

**Figure 3:**
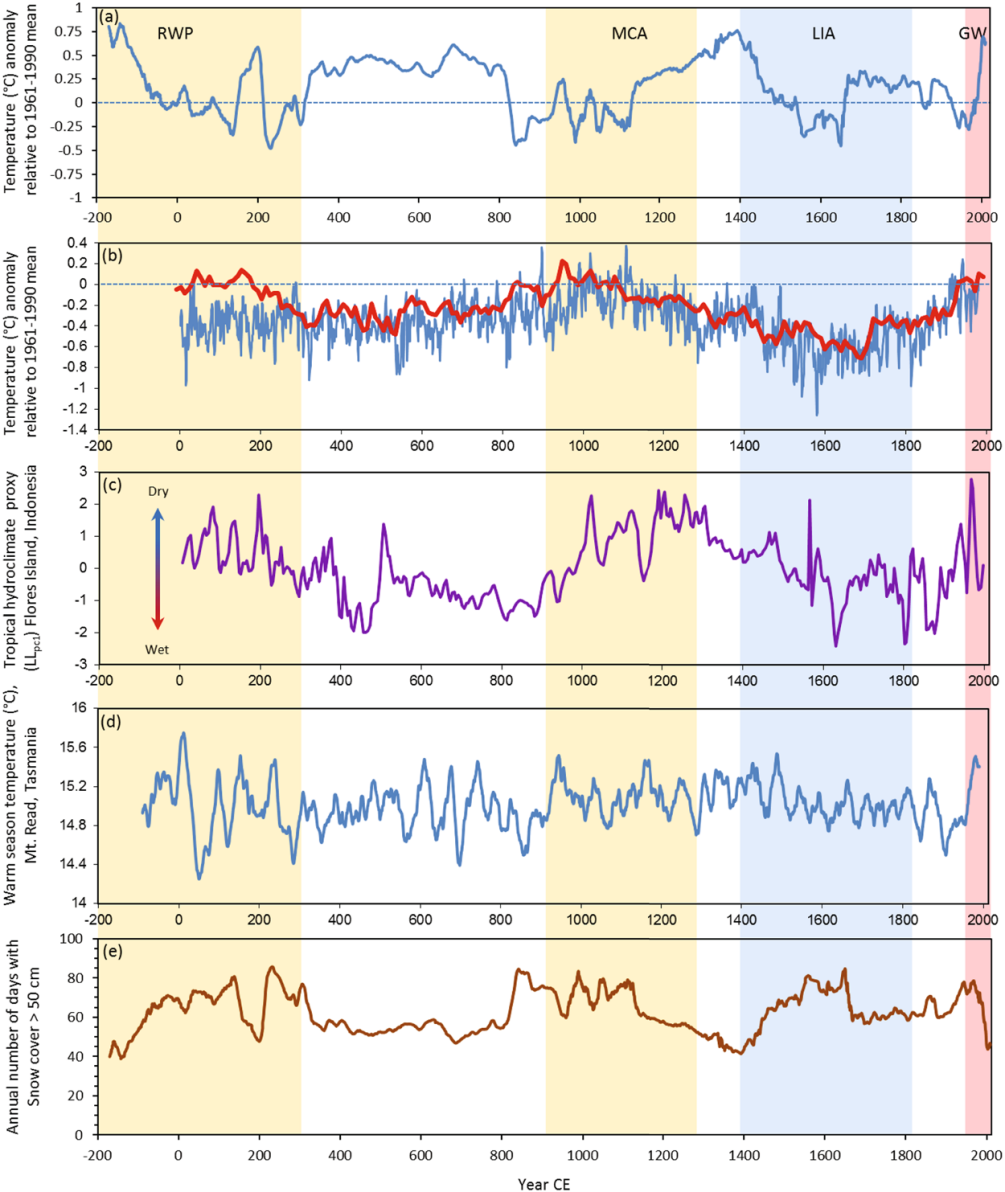
Reconstructed Yarrangobilly Caves Tmax (5 yr mean) for the Australian alpine area showing departure from the 1961–1990 mean (**a**); reconstructed Northern Hemisphere temperatures and associated departure from the 1961–1990 mean blue line^40^ and red line^41^ (**b**); tropical hydroclimate variability for the western Pacific using the proxy LL_PC1_^42^ (**c**); warm season temperature reconstruction for Mt. Read, Tasmania based on Huon pine growth rings^43^ (**d**); annual number of days with snow cover >50 cm at Deep Creek, Snowy Mountains, Australia (**e**).

Our δ^13^C derived Yarrangobilly Caves paleo-temperature record for annual mean Tmax Fig. 3a), shows onset of cooling signalling the end of the RWP around 140 BCE. Tmax was approximately 0.8 °C above the Tmax mean for the reference period 1961–1990 CE, and snow cover was near modern minimums (Fig. 3e). A cooling trend then continued for approximately 300 years until around 130 CE when Tmax increased over a 65 year period before decreasing rapidly. From here through the period referred to in the Northern Hemisphere as the Dark Ages Cold Period (DACP), there are marked milder conditions through to a rapid cooling event around 810 CE. This was followed by a period of variable but mostly cooler temperatures and increased snow cover for around 300 yrs including the Oort Minimum, after which Tmax temperature increased and snow cover declined from around 1100 CE (Fig. 3a and e).

A warming trend spanning the period from approximately 1130 CE to 1392 CE, overlaps with the widely reported timing of the MCA from Northern Hemisphere sites, although a clear synchronous onset of the MCA around the mid to late 900 s CE as seen in Northern Hemisphere records^44,45^ is not evident (Fig. 3a,b). This warm period in the Australian alpine region corresponds well to that derived primarily from tree rings in Tasmania (and New Zealand) before a gradual transition in temperatures to the colder LIA over a 200 year period^46^. This is followed by a rapid cooling event coeval with the Maunder Minimum, with the coldest temperatures in the record around 1650 CE (Fig. 3a).

Our reconstructed snow cover record (Fig. 3e), shows a similar but inverse pattern to temperature with the lowest number of days with snow cover greater than 50 cm deep, occurring coeval with periods of higher annual mean Tmax values (Fig. 3a). Colder temperatures indicated by our Tmax record led to much more persistent seasonal snow cover than present between 1500 CE to 1700 CE reflecting the impact and timing of the LIA in this region. Reconstructed Tmax temperatures then increased from around 1660 CE remaining above the 1961–1990 CE reference period for approximately the next 200 years with a slight cooling trend toward the first half of the 20^th^ Century (Fig. 3a). Increasing Tmax temperatures since the late 1960s are coeval with a marked decrease in the numbers of days with snow cover greater than 50 cm deep. When evaluated against the reconstruction, snow course records suggest that cover is now at the lowest for at least the past 2000 years (Fig. 3e).

### Placing recent global warming in context

Placing recent warming and change in seasonal snow cover in the Australian Alps in context against anthropogenic and long term natural climate variability, is essential to predict hydroclimate. The resolution of this Southern Hemisphere record also provides guidance on the global synchronicity, timing and magnitude of major climate events known to impact people and environments across the past 2000 years.

Our record suggests the Australian snowpack is now at a 2,000 year low, while reconstructed alpine Tmax temperatures are beginning to approach Tmax values around the height of the RWP at 150 BCE and MCA 1390 CE. Observed annual Tmax values are only now exceeding our reconstructed Tmax for the past 2000 years. The rate of temperature increase from 1970 to 2005 was approximately 2.6 times faster than the fastest rate of warming during the MCA.

The variability in our record highlights local scale influences, most notably topography which can amplify the effects of even subtle changes in atmospheric circulation through change in cloud cover, precipitation and wind. Accordingly, paleoclimate reconstructions as we present here provide valuable insight to the magnitude and rate of change of local scale climate which is often masked in larger scale composite paleo-temperature reconstructions. The reconstructed Yarrangobilly Caves record shows significantly more variability in temperature over the past 2000 years than for example, composite Northern Hemisphere mid-latitude temperature reconstructions (Fig. 3b) but is similar to the warm season temperature reconstruction for Mt Read, Tasmania (Fig. 3d).

In general our alpine record shows warmer/colder periods in the Northern Hemisphere correspond to colder/warmer Tmax conditions in the Australian alpine region, and wetter/drier conditions in the tropical western Pacific (Fig. 3a–c); indicative of the well documented biopolar seesaw effect^47^. The temperature reconstructions presented in Fig. 3a,b only then begin to converge during the height of LIA and around the timing of the Maunder Minimum with temperatures well below the 1961–90 means in the Australian alpine record and Northern Hemisphere records, while snow cover is near maximum values. This colder period corresponds to wetter conditions in the western tropical Pacific (Fig. 3c), possibly analogous to conditions reported during Heinrich Events in the tropics^48,49^, while the signal of the LIA in the Mt Read record (Fig. 3d) is subtle.

Following 150 BCE, temperatures in the Australian alpine area decreased with this temperature signal coeval with multiple glacial advances in the Southern Alps, New Zealand^50^ and overlapping with evidence of increased climate variability and prevalence of colder winters and drought across much of the Northern Hemisphere^1,3^ referred to as the Dark Ages Cold Period (DACP)^51–53^. Our record shows rapid decreases in Tmax around 200 and 800 CE followed by variable conditions for the ensuing 300 years. Overall, from around 450 CE to 795 CE mean annual Tmax temperatures were up to 0.6 °C above the 1961–90 mean in the Australian alpine area. This corresponds to a period in the Northern Hemisphere mid-latitudes when temperatures were below 1961–90 means (Fig. 3b), and the western tropical Pacific hydroclimate was more active (wetter) (Fig. 3c) similar to the LIA. Elsewhere, the DACP period is notable for known rapid transitions in wet to dry conditions identified as contributing to pre-Classic abandonment (c.150 to 250 CE) and Terminal Collapse (c.810 to 910 CE) of the Maya civilisation^54^. Our record supports an interpretation of the DACP as a time of non-synchronous global response characterised by decadal-scale rapid warm/cool and wet/dry transitions^51,55^. In the Australian alpine area rapid transitions between warm and cool periods we believe are linked to changes in prevailing circumpolar westerly flow and its inaction with the mountainous topography of south-eastern Australia including Yarrangobilly Caves.

Evidence of a warm MCA (≈1100 CE to 1390 CE) in the Australian alpine region (Fig. 3a) is aligned with records from New Zealand spanning this period which indicate warmer and drier conditions over the western South Island, likely caused by increased blocking of frontal systems^50^. These weather patterns support anomalous east to north-easterly winds over New Zealand and south-eastern Australia, and are thought to have assisted Polynesian migrations into the southwest Pacific at this time^56^. Our reconstructed Tmax for these warmer conditions peaks around 1390 CE at + 0.8 °C above the 1961–90 mean, similar to the peak Tmax during the RWP. These results are aligned with the findings that show the period from 1150 to 1350 CE to be the warmest pre-industrial chronzone of the past 1000 yrs for southeast Australia^46^.

The warmth in the Australian alpine area corresponding to the MCA was followed by a cooling trend coeval with the LIA widely reported in temperature reconstructions for the Northern Hemisphere^40,41,57^ and seen in New Zealand palaeoclimate archives also before onset of very cold conditions through 1600 to 1700 CE^58^. This period has been linked to increased southerly airflow^50,56^, solar variability and volcanic forcing of climate^57,59^. These 300 years of regional Southern Hemisphere cooling is coeval with evidence of widespread colder temperatures across the Northern Hemisphere, and supports a globally synchronous LIA cooling of climate.

The coldest period of our reconstructed temperature record corresponds to the Maunder Minimum around 1650 CE before onset of a prolonged, albeit a subdued warming trend extending too early to mid-20^th^ Century after which rapid warming linked to global warming is evident. This warming trend is seen in the Mt Read record (Fig. 3d) also and two regional Northern Hemisphere temperature reconstructions (Fig. 3b). The corresponding proxy hydroclimate record for the western Pacific tropics displays a drying trend at this time (Fig. 3c).

Throughout our reconstructed Tmax record there are distinct periods of rapid warming (cooling), that briefly approach rates of 0.1–0.15 °C per decade including around 145 (200), 250 (820), 910 (960) and 1130 (1540) CE. By contrast, warming since 1970 has accelerated to an average rate of approximately +0.4 °C per decade in our record, the fastest rate of warming by both magnitude and sustained duration experienced in the last two millennia.

Rapid cooling events and increased snow cover in our record (Fig. 3e) coincide with known major Southern Hemisphere volcanic eruptions including a series of large south American eruptions from Calbuco and Melimoyu and from the Taupo region in New Zealand (~200–260 CE); Dakataua, Papua New Guinea and Mt Taranaki, New Zealand (800–820 CE); Paektu Mountain, Eastern China (945–984 CE); Minchinmávida, Chile (1541–1550 EC) and Tambora (1815 CE)^60^. The absence of strong signatures from eruptions such as Samalas (1257 CE) is not unexpected as impact on climate, and therefore preservation in a geologic archive, is dependent on the seasonal timing of the eruption, geographic location, teleconnections and prevailing meteorology^61,62^. Seasonal timing of large eruptions may be particularly important with high latitude summer eruptions shown to cause strong hemispheric cooling^63^.

Our reconstructed snow cover record for the past 2000 yrs is the first near annual snow cover record and highlights the potential of our approach to obtain insight to snow cover variability. The coherent yet opposite sign of the reconstructed snow cover record compared to Tmax is not unexpected given the high dependence of snow cover on temperature, particularly in temperate marginal alpine climates. This relationship is caused by change in temperature across the freezing point causing more precipitation to fall as rain as temperature increases reducing overall snowfall, and increasing melt of existing snow cover. Recent rapid decrease in our reconstructed snow cover record (Fig. 3e) over the past 5 decades is at least an order of magnitude greater than for similar periods over the past 2000 yrs and is driven by corresponding global warming. This effect is being compounded by change in atmospheric circulation that has seen a reduction in winter snowfall associated with the passage of cold fronts, and an increase in rainfall events with moisture sourced from the tropical seas northwest of Australia^22^. Accordingly, the recent rapid decrease in seasonal snow cover in our record is likely to continue and result in dramatic change in the hydrology of the Australian Alps and rivers which drain from this region.

Rapid change in temperature and associated snow cover over the past fifty years is similar to that reported elsewhere for temperate alpine areas including the western United States of America and Europe, and is attributed to global warming^64,65^. Impacts include increased flood risk due to more frequent rain on snow events, increased water stresses as seasonal snow packs decrease, loss of winter snow sport opportunities as both spatial extent and duration of snow cover rapidly declines, and change to snow dependent ecosystems. Results confirm that speleothems and carbon isotopes offer an excellent archive of palaeo-data on temperature and snow cover variability (particularly in non-water limited settings) which may be used to greatly extend limited snow course observations back to pre-industrial periods. Such research will give insight to variability of alpine hydrometeorology essential to plan for future sustainable alpine water resources in a changing and warming climate.

## Methods

### Stable isotope analysis and age model development

Stable isotope analysis (δ18O and δ13C) was conducted by acid digestion using an individual vial acid drop ThemoScientific Kiel IV carbonate device interfaced to ThermoScientific MAT-253 dual-inlet isotope ratio mass spectrometer at the University of California, Santa Cruz, USA and Nanjing University, Nanjing, China. Ratios listed in Table S1 for the 11 samples used for developing the geochronology of JC001 refer to activity ratios normalized to the corresponding ratios measured for the secular-equilibrium HU-1 standard. ^230^Th ages are calculated using Isoplot/Ex 3.75^53^. Non-radiogenic ^230^Th correction was applied assuming non-radiogenic ^230^Th/^232^Th atomic ratio = 4.4 ± 2.2 × 10^−6^ (bulk-earth value), and ^238^U, ^234^U, ^232^Th and ^230^Th are in secular equilibrium. Non-radiogenic ^230^Th correction results in large age error magnification for samples with low ^230^Th/^232^Th ratios.

Using the 11 dates presented in Table S1, the age model for JC001 was developed using the StalAge algorithm^66,67^. An age of 2012 (−62 BP) was used for the tip of the stalagmite as this was the date it was removed. The age and 95%-confidence limits were calculated in StalAge with Monte-Carlo simulations. Here the final age model was determined through an ensemble of 30 iterations of the algorithm to ensure a representative age model using ensemble mean values (Supplementary material Table S2).

### Linear Models

To obtain a mean annual value from the time varying stable isotope record (1.03–1.6 years), each record was interpolated at a 1/7 year resolution and averaged over a calendar year^27^. A 5 year simple moving average was applied to the interpolated annual δ^18^O and δ^13^C records, and instrumental climate records of temperature and snow days of depth ≥ 50 cm for the calibration of a linear model. The rate of temperature change was calculated by smoothing the instantaneous rate of change between each member of the temperature reconstruction over the preceding five decades.

## Electronic supplementary material

Supplementary Information
