# Supplementary material for: Global warming in the context of 2000 years of Australian alpine temperature and snow cover
Source: Sci Rep. 2018 Mar 13;8:4394. doi: 10.1038/s41598-018-22766-z (PMC5849736; doi:10.1038/s41598-018-22766-z)
Supplement: Supplementary file 1 — Supplementary Information [file 41598_2018_22766_MOESM1_ESM.doc]

Supplementary Information for

Global warming in the context of 2000 years of Australian alpine temperature and snow cover

Hamish McGowan^1^, John Nikolaus Callow^2^, Joshua Soderholm^1^, Gavan McGrath^2^, Micheline Campbell^2^, Jian-xin Zhao^3^

^1^Atmospheric Observations Research Group, School of Earth and Environmental Sciences, The University of Queensland, Brisbane, Australia 4072.

^2^UWA School of Agriculture and Environment, The University of Western Australia, Perth, Australia 6009.

^3^Radiogenic Isotope Facility, School of Earth and Environmental Sciences, The University of Queensland, St. Brisbane, Australia 4072.

**Introduction**

This supporting information provides:

Table S1 presents details of the eleven uranium series dates obtained for stalagmite JC001. The age model used to establish the 2000 year reconstructed temperature and snow cover series was developed using the StalAge algorithm of *Scholz and Hoffmann,* [2011]. Ensemble means for the 11 dates and their ± 2δ output from the StalAge algorithm are presented in Table S2 and shown graphically in Figure S1. Plots of annual mean maximum temperatures (5 year means) and annual number of days snow cover ≥ 0.5 m for the alpine locality of Yarrangobilly Caves and locality of Deep Creek respectively are presented in Figure S2. Also shown are the linear functions developed from this data used to calculate paleo-temperatures and snow cover and their associated r-squared values and RMSE.

**Table S1:** Uranium series dating results for 11 sub-samples milled from stalagmite JC001 with ages shown in years BP (1950) with ± 2σ, and respective year CE or BCE

Note: Ratios listed in the table refer to activity ratios normalized to the corresponding ratios measured for the secular-equilibrium HU-1 standard. ^230^Th ages are calculated using Isoplot/Ex 3.75 [*Ludwig,* 2012], using decay constants of *Cheng et al.,* [2000]. Non-radiogenic ^230^Th correction was applied assuming non-radiogenic 230Th/232Th atomic ratio = 4.4±2.2 x 10^-6^ (bulk-earth value), and ^238^U, ^234^U, ^232^Th and ^230^Th are in secular equilibrium. Non-radiogenic ^230^Th correction results in large age error magnification for samples with low ^230^Th/^232^Th ratios. uncorr. and corr. denote uncorrected and corrected. BP refers to before present where present = 1950AD. CE and BCE denote Common Era and before Common Era, respectively.

**Table S2**. Presented age limits are the mean of a 30 iteration ensemble. Standard 2δ are also presented below. The bold values represent the collection date of JC001, i.e. 2012 referenced to 1950.

| Depth | corr. ^230^Th Age (BP) | ±2δ |
| --- | --- | --- |
| **0** | **-62.000000** | **0.01** |
| 5 | 56.761267 | 240.464 |
| 17 | 613.794235 | 228.16 |
| 38 | 943.651869 | 60.553 |
| 55 | 1538.821271 | 85.985 |
| 72 | 2042.764120 | 93.205 |
| 90 | 2285.978025 | 68.218 |
| 107 | 2730.711450 | 35.000 |
| 171 | 4031.685929 | 67.865 |
| 235 | 4805.123404 | 151.530 |
| 285 | 5685.784835 | 67.424 |
| 345 | 6631.532285 | 59.763 |


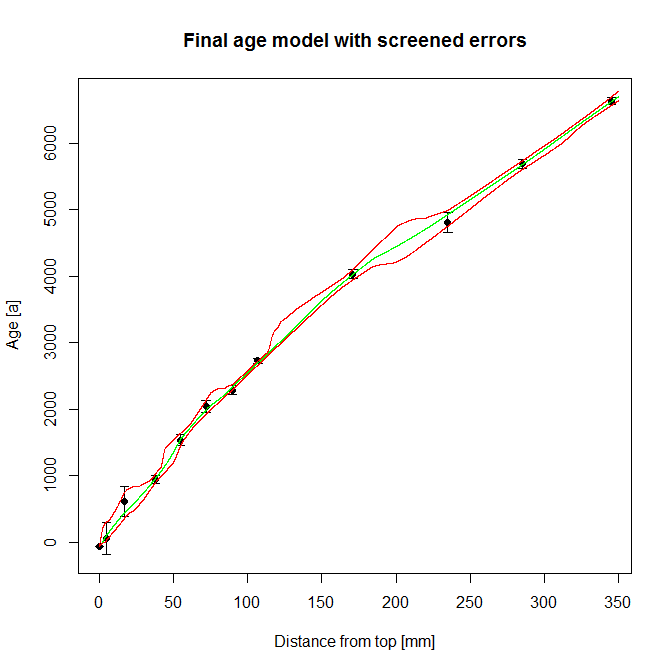


Figure S1. Final age model (green line) for JC001 developed using the StalAge algorithm [*Scholz and Hoffmann,* 2011] for the entire length of the stalagmite with 95%-confidence limits shown in red.

(a)


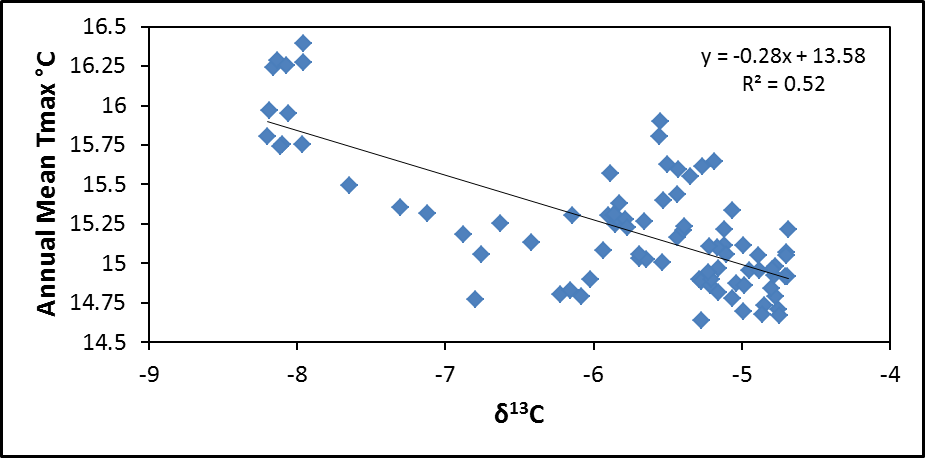


(b)


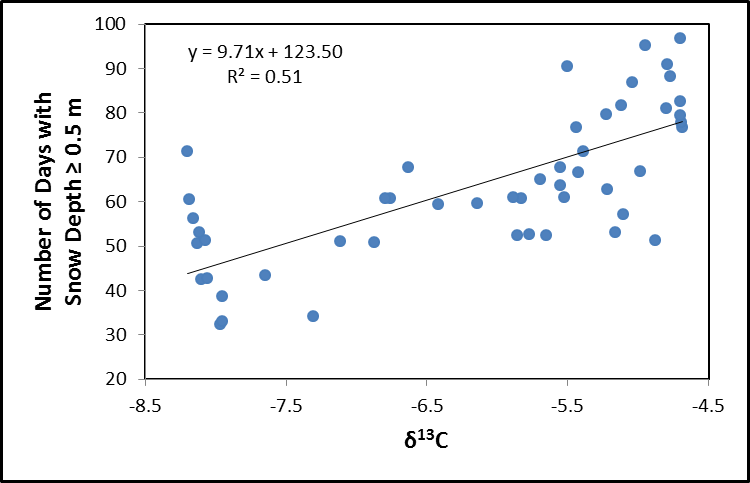


**Figure S2**. Plots of 5 year simple moving averages of annual mean Tmax values for the alpine locality of Yarrangobilly Caves (a), and number of days each winter snow cover exceeded 50 cm at Deep Creek Snow Course (b) against corresponding JC001 δ^13^C values. Also shown are the linear functions used to reconstruction Tmax and snow cover and their respective r-squared values. The corresponding RMSE for the observed against modelled data for Tmax (1911 – 2013) is 0.28 °C and for days snow cover > 50cm (1959 – 2009) is 31.9 days.
